# Artificial or intelligent? Machine learning and medical selection: possibilities and risks

**DOI:** 10.15694/mep.2018.0000256.1

**Published:** 2018-11-13

**Authors:** Paul Tiffin, Lewis Paton

**Affiliations:** 1 University of York

**Keywords:** machine learning, artificial intelligence, personnel selection, medical selection, logistic regression, XGBoost

## Abstract

This article was migrated. The article was marked as recommended.

Machine learning approaches form the basis of “artificial intelligence” and have been increasingly applied in health services settings. It has been shown that such approaches may produce more accurate predictions in some contexts, compared to conventional statistical approaches, and may also reduce the costs of decision-making through automation.  Nevertheless, there are both general limitations to developing and implementing machine learning approaches that must be borne in mind. To date, relatively little research has been published on the potential for machine learning to support personnel selection. Moreover, there are particular challenges and issues that need to be considered if such methods are to be used to support decision-making in medical selection scenarios. This article describes some of these potential advantages and challenges and presents an illustrative example, based on real-world data, related to the selection of medical undergraduates.

## Introduction

There has been much publicity about the possibilities for ‘artificial intelligence’ to change our lives. Opinions have often been polarised with, on one hand, some promoting the idea that artificial intelligence, in the context of advancing robotics, will free mankind from the drudgery of all manual labour, ushering in a utopia of leisure and pleasure. On the other hand the late cosmologist, Stephen Hawking, famously stated that ‘ *the development of full artificial intelligence could spell the end of the human race*’. Machine learning, the basis of artificial intelligence, occurs when a system learns from novel information presented to it in order to complete a particular task. Such learning is often classified into “supervised” and “unsupervised”. In unsupervised learning the task is usually to cluster or classify observations without reference to a particular ‘target’ or outcome. An example of such an approach would be ‘shopping basket analyses’ which attempt to predict which retail items tend to be brought together by customers. In contrast, in supervised learning the machine is fed a series of examples in order to allow it to learn how to link predictors (or ‘features’) to a specific outcome (or ‘target’). Ideally such learning should generalise so that when the system is shown an unfamiliar dataset the machine will be able to accurately predict the new (unseen) outcomes. Most of the recent examples of machine learning in healthcare settings have been based on such an approach. For example, the ability of a system developed by DeepMind (formerly part of Google) to automate the diagnosis of eye disease from medical images ( De Fauw *et al.*, 2018). In theory, such systems only need to be as accurate as human doctors in order to justify their implementation, as they will free up medical staff time, providing cost savings. However, in practice it may be that misdiagnosis by a machine is much less acceptable than by a human clinician. This may be a component of ‘algorithm aversion’ ( Dietvorst, 2016), whereby machines are viewed more negatively for making the same mistakes as people. Indeed, fallibility is often considered a key part of being human, with Seneca the Younger famously quoted as stating that *errare humanum est* [‘to err is human’]. This may be one of the reasons that, in practice, the suggestions of decision support tools are often overridden by clinicians ( Roshanov *et al.*, 2013). Despite the hype surrounding artificial intelligence there are still relatively few examples of the approach being fully implemented as part of routine clinical services, though there are calls to make an understanding of the principles of artificial intelligence a core requirement of medical education, in preparation for its widespread utilisation ( Wartman and Combs, 2018). One possible hindrance to the use of such algorithms in practice, when used to make diagnostic or treatment decisions, is that they are effectively medical devices. As such they are subject to stringent regulations in most jurisdictions and considerable resources are required in order to satisfy these so that they can be legally used in practice.

The quality of the medical workforce often determines the quality of clinical outcomes and patient experience. Thus, staff selection methods could also be considered a health technology. Outside of medicine, machine learning algorithms are already being used in personnel selection decisions. However, possibly due to commercial sensitivity, relatively little has been published to date on the potential application of artificial intelligence when recruiting and appointing staff. As might be expected, Google, via their ‘People Analytics’ department, have started using machine learning to inform their personnel selection decisions, as well as to improve retention rates ( Shweta, Kritika and Anupama, 2018). Machine learning may also offer possible solutions to specific staff selection issues; for example, by circumventing the need for expert scoring keys for situational judgement tests via simply predicting outcomes (such as future supervisor appraisals) from a candidate’s raw test response patterns ( Guenole, Weekley and Ro, 2016). Machine learning has also been applied to the scoring of postgraduate assessments in medical education, though validation studies were often lacking ( Dias, Gupta and Yule, 2018). Moreover, what is noticeably absent from the scientific literature is robust empirical evidence that artificial intelligence leads to the selection of a more effective and productive workforce, compared to conventional methods.

## The potential strengths and limitations of machine learning

The term ‘machine learning’ applies to a broad range of methods, though many share similar mathematical underpinnings to conventional statistical approaches. Tradiotional statistical methods usually aim to produce *explanatory* models. That is, the proportion of the variance in a particular outcome variable that can be explained by the values of one or more predictors. Such explanatory models help us understand the relationship between the predictors and outcomes, and ideally support theories of causation that can be further tested. An example may be modelling the relationship between the scores on different subscales of an aptitude test and subsequent academic performance in medical school. The findings of such studies may help us comprehend the link between different facets of cognitive functioning and the various aspects of undergraduate academic performance. In contrast the focus of machine learning is *prediction*, rather than explanation. Indeed, machine learning algorithms have previously been described as pursuing a predictive task *“..with all the relentlessness of a T-101 terminator pursuing Sarah Connor through a Los Angeles police station..”* ( Tiffin and Paton, 2018). Compared to conventional statistics machine learning can take a more flexible approach to modelling the relationship between predictors and outcomes and can often better capture complex, non-linear relationships. Moreover, via the ‘brute force’ that modern computing can offer, a machine can iteratively try thousands, or even millions, of permutations of a model in order to derive the most accurate prediction of the target from the ‘features’ (predictors). Indeed, ‘ensemble methods’ may be used to build numerous models then combine the predictions from each in a way which improves accuracy when faced with a novel dataset. This approach could be visualised as the models in the ensemble represented by piranhas in a shoal, each nibbling a different part of an animal (the prediction problem) in order to strip the carcass as efficiently as possible. Thus, it is unsurprising that, in terms of outright prediction, machine learning often outperforms conventional statistical approaches.

However, such accuracy comes with a number of associated costs. Firstly, machine learning algorithms are so effective at linking predictors to outcomes that there is a risk of ‘overfitting’. ‘Overfitting’ occurs when a model is inadvertently fitted to the noise (or error) rather than the underlying signal. Such an overfitting model seems to describe and fit the ‘training data’ from which it was derived, almost perfectly. However, when the model is applied to a separate, fresh dataset it demonstrates little predictive ability. A good analogy from tailoring would be having a bespoke suit made for a particular individual that provides a perfect fit. However, the clothes, when worn by anyone else, would be embarrassingly ill fitting. Consequently, considerable efforts have been made to counter this issue using various methods. Secondly, in general, the more complex (and often most effective) machine learning approaches do not give rise to interpretable models. That is, they are able to accurately predict an outcome from a set of predictors, even from data that they have never encountered before, but it is not possible to understand how they got there! It is for this reason that machine learning models are sometimes described as ‘black boxes’ with no one knowing what goes on inside. This may be a particular issue in personnel selection. A candidate may understandably want to know the reason that they were unsuccessful at a job application. If the decision was substantially or wholly based on the recommendation of a machine learning algorithm the organisation may well not know themselves! Being unable to justify such a high stakes decision could actually breach employment legislation in a number of jurisdictions. Moreover, ethically, it is not always clear where the responsibility for the performance and behaviour of such algorithms lie as they are constructed and implemented by numerous actors including designers, end-users and developers of both the hardware and software required. This issue has been termed ‘distributed agency’ that may need to be addressed by novel moral and legal frameworks ( Taddeo and Floridi, 2018).

Machine learning models are only as good as the data on which they are trained. Thus a suitable quantity and quality of information relating to potential predictors (features) and outcomes must be available. Deficiencies with either can lead to several notable problems in practice. Firstly, obtaining a ‘hard’ (objective) outcome to train an algorithm against can be challenging in personnel selection. For instance, ratings of work-based performance are only available in those candidates selected. Also, measuring this construct usually relies on relatively subjective approaches, such as supervisor ratings. At worst the resulting machine learning algorithms may actually exaggerate the human biases that they were intended to overcome. Also, if a particular outcome is relatively rare (e.g. disciplinary proceedings) then a machine may achieve a good ability to predict its absence (i.e. ruling the event out) but not its likely occurrence. This is often an artefact of the optimisation process, by which accuracy is maximised during algorithm training- it is relatively easy to achieve high accuracy merely by predicting the absence of a rare outcome in most or every case. For instance, imagine a situation whereby one had to predict future disciplinary issues in a set of medical students, which occurred in 2% of the sample over five years. By predicting an absence of disciplinary problems in every case one would achieve 98% accuracy without effort. There are ways to mitigate against this effect (see motivating example, later). Secondly, the group of individuals from which data are drawn for training may not be representative of wider populations, leading to poor generalisability and potential bias. Well publicised examples of such issues include ethnic (racial) bias in algorithms predicting the likely location of crimes and the risk of re-offending in prisoners as well as the poor performance of some facial recognition software for non-Caucasians ( Cossins, 2018). As these models are often ‘black boxes’ it is often not clear under what circumstances they may be invalid, or lose predictive ability, through changing trends. This could particularly affect staff selection algorithms as changes to the structure and standards of educational qualifications could render new data ‘out of sample’. One way of addressing such issues may be to repeat validation exercises periodically to ensure that such models remained acceptably accurate over time and settings. It may be, as in healthcare, a blended approach is required, where machines are used to support human decisions, rather than over-ride them. Thus, it may also be possible to combine data analytics with more traditional approaches, such as interviews. Thus, machines may be able to help select candidates for interview and support the focussing of the interview on the most relevant topics. At least one company currently promotes such hybrid approaches ( Clearfit, 2018).

In order to illustrate some of the potential pros and cons of machine learning applied to medical selection we present a motivating example.

## Machine learning and medical selection- an illustrative example: Predicting academic performance in the pre-clinical years

One of the desirable qualities in medical school applicants is academic ability, which helps ensure that the candidate will be able to cope with the intellectual demands of their undergraduate and postgraduate studies. In the UK, and elsewhere, most medical students who have to leave the course academic reasons do so in the first two years of study, with relatively little attrition after this point. Moreover, medical schools tend to be keen to avoid having to host resit exams, as these absorb relatively large quantities of resource for a relatively small number of initially unsuccessful students. Thus, when confronted by two apparently similar candidates it could be useful to know, from the routine information available on both, what the likely probability is that they will pass both year one and year two without the need for any resits, or indeed without needing to leave the course for academic reasons. Such an algorithm could support making a decision in such a high-stakes situation. However, this is extremely challenging prediction problem. Firstly, in the UK, and often elsewhere, failure at end of year exams is a relatively uncommon outcome. This makes modelling relatively sparse events challenging (see earlier). Secondly, medical school applicants are relatively homogenous with high predicted, or achieved, school grades and cognitive functioning, as estimated via commonly used aptitude tests. This homogeneity is even more marked in those who have successfully entered medical school. Thus, with such little variance amongst individuals we are dealing with a relatively “information poor environment”. This makes prediction even more challenging. Finally, it is well known that medical schools may have varying academic standards ( Devine, Harborne and McManus, 2015) but a selection team wants to know what the odds of an applicant failing *their* particular course is. Thus, any model must take into account this variation by medical school. Using data used in a previously published study which employed conventional statistical modelling ( Mwandigha *et al.*, 2018) we aim to show how machine learning can provide some advantage in such challenging prediction scenarios, showcasing some of the ‘tricks’ that can be employed by these methods.

## Data

The data used was routinely arising information recorded as part of UK medical selection processes. A dataset consisted of a subset of previously analysed information on UK medical applicants. In this case we took a subset of 6108 medical entrants who had information relating to the academic outcomes from both year one and year two of their undergraduate studies (i.e. the preclinical years). That is, whether the student had passed both years at first sitting, required a resit or had to resit the whole year of study. For the purposes of this analysis the outcome was dichotomised into ‘passed both years at first attempt’ or ‘required at least one resit’.

Data on the students’ previous academic achievement at school, in terms of both GCSE and Advanced (A) level examinations were available for students from England, as was the overall secondary (high) school performance of the educational institution attended at the time of application. Sociodemographic information was also available on reported ethnicity, socioeconomic status and type of school previously attended. UK Clinical Aptitude Test (UKCAT) performance at first attempt was also available. The data were managed in a similar way to a previous study ( Mwandigha *et al.*, 2018), with some minor modifications to the data ‘pre-processing’ used to accommodate the machine learning process. Also incorporated into the models were the average UKCAT scores achieved by the candidate’s peers at that particular medical school in that year. Thus, this was incorporated into the modelling as a medical school-level variable, in an attempt to adjust for some of the variation for academic standards across universities.

## The Machine Learning method: Extreme Gradient Boosting

We used a machine learning approach and compared it with a traditional, stepwise logistic regression model. The machine learning method we used is known as ‘Extreme Gradient Boosting’ as implemented in the *XGBoost* R package ( Chen and Guestrin, 2016). The method was selected as it is known to work well even with small and medium-sized datasets (i.e. several hundred to several thousand observations). Extreme gradient boosting combines a number of methodological approaches to prediction; the use of decision trees; ‘ensembling’- where numerous slightly differing models are created, and the results averaged or voted on, and; ‘boosting’ where the algorithm successively focuses on the observations where the outcome is increasingly difficult to predict. By combining all three approaches, overall, extreme gradient boosting tends to outperform algorithms which only use one or two of these methods. This is evidenced by its common use in winning entries to machine learning ‘Kaggle’ competitions, where data scientists vie with each other to produce the most predictive algorithms for certain datasets ( Kaggle Forum, 2016).

## Model building

**Figure 1.**
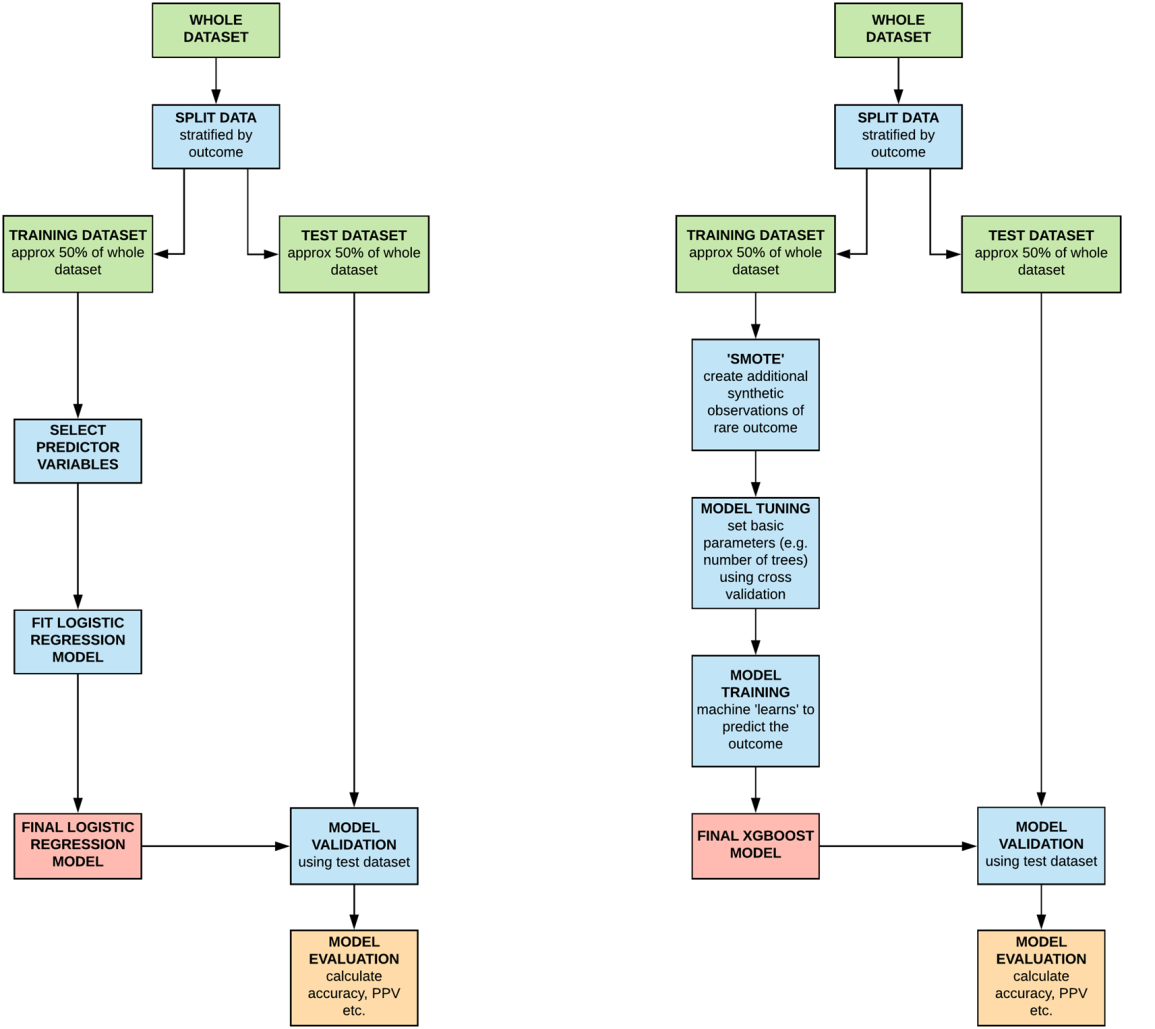
Flow diagram illustrating the modelling building process for both the machine learning (‘extreme gradient boosting’ [XGBOOST’]) and traditional (logistic regression) methods.

It is usual practice to divide datasets into *training* and *test* datasets when developing machine learning algorithms, although numerous approaches to dividing up data exist, depending on the scenario and availability of data. This is so that a model can be developed on the first (training) dataset and validated on the separate, ‘held back’ test dataset. Almost invariably models developed on the training set predict the outcome almost perfectly but when tested on the ‘heldback’ dataset demonstrate poorer, though hopefully still acceptable accuracy. The model building process is shown in figure 1. Note that, in this case, because the outcome of interest (exam failure) was relatively uncommon, test and training datasets were created by randomly dividing the data in two, though candidates who had failed at least one year had an equal probability of being in either test or training dataset. To help the algorithm train to predict the less common outcome (exam failure) we used the SMOTE package in R to create ‘synthetic students’, based on the real ones who had failed an exam at first sitting, to balance the outcomes in the training data set. Missing observations were filled in using a single imputation using the Amelia package in R. There was also a ‘tuning’ phase for the machine learning, where the basic model settings were altered (e.g. the number of decision trees ‘grown’ each time) to optimise its predictive performance in the training dataset. By randomly splitting the data, selecting predictor variables to include and imputing missing values and so on, we obviously introduced elements of chance into the results from each modelling run. Therefore the process of model building for both for the logistic regression and the machine learning algorithm was repeated 1000 times so that the overall results could be averaged and any variation quantified.

## Results

**Figure 2.**
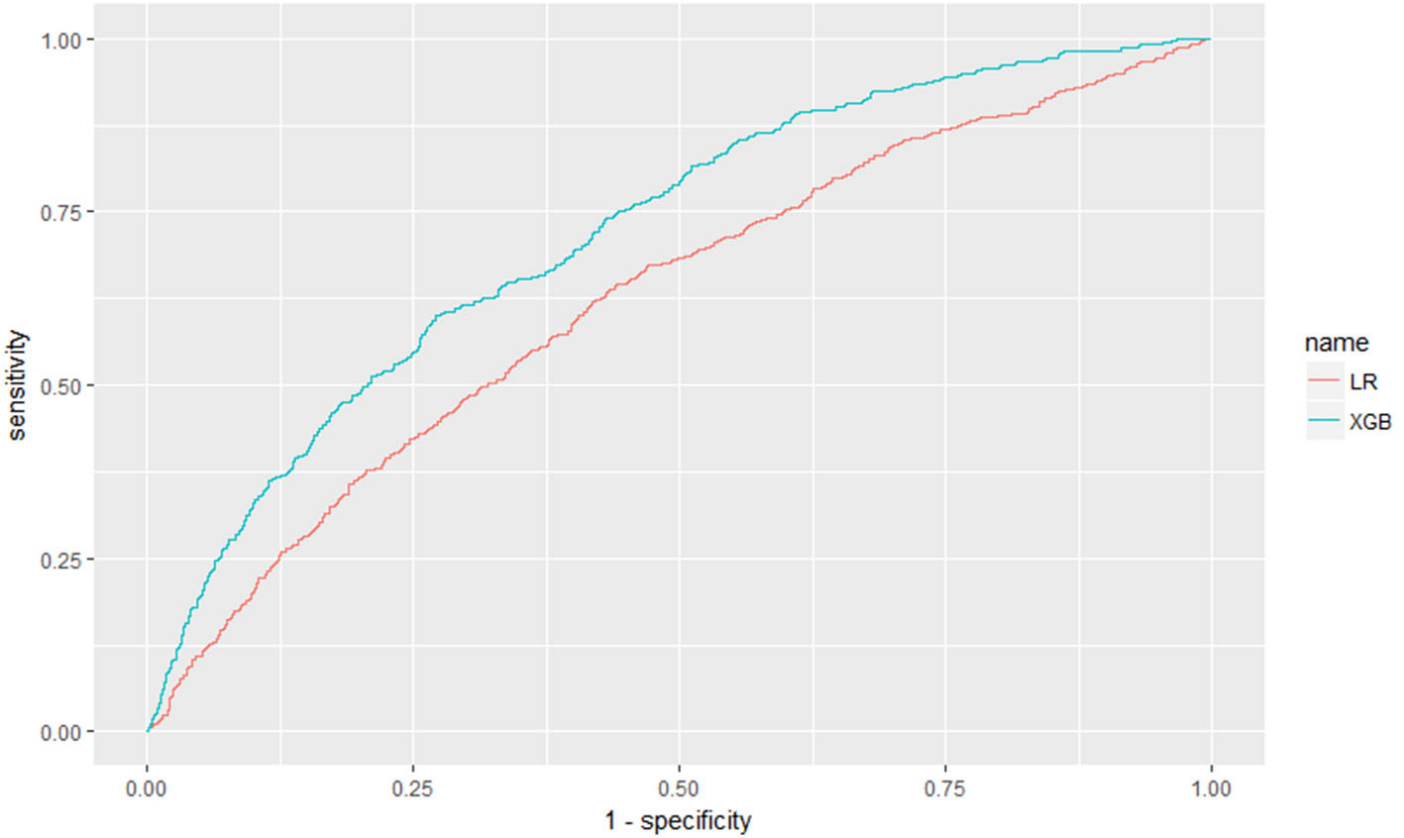
The Receiver Operator Characteristic (ROC) Curves for the predictions for passing the first two years of medical school at first sitting for the logistic regression (LR) and machine learning (XGB) models.

The potential of an assessment diagnostic process as a screening test is indicated by the ‘area under the curve’ (AUC) of the Receiver Operator Characteristic (ROC) curves, that ideally should be greater than 0.5 (on average no better than chance) and as close to 1 as possible (i.e. perfect prediction). ROC curves for the traditional, logistic regression model-based prediction and the machine learning predictions are shown in figure 2. These show the ability of the models to predict which students are likely to pass the first two years of medical school, for differing hypothetical cut-scores. These ‘scores’ are actually estimated probabilities, from the models, for an entrant passing their exams at first sitting. Other important indices for appraising the performance of a predictive or screening test are Positive Predictive Value (PPV- the proportion of individuals that ‘screen positive’ that are ‘true cases’), Negative Predictive Value (NPV- the proportion of individuals that ‘screen negative’ that are ‘true non-cases’), sensitivity (the ability to detect ‘true cases’) and specificity (the ability of a test to rule out ‘caseness’). In this situation we define a ‘case’ as a student who passes their exams at first attempt.

The mean of these values for both modelling approaches are shown in table 1. As can be seen in the table, the AUC for the machine learning approach was significantly greater than that for the logistic regression, highlighting the superior predictive ability. Aside from this there were a number of striking differences in performance between the logistic regression and the machine learning approach. Firstly, the sensitivity of the logistic regression model was higher than that for the machine learning algorithm. In effect what this meant was that the logistic regression was able to almost perfectly predict which entrance were likely to pass both end of year exams at first sitting (mean sensitivity 99.7%). In this regard the machine learning approach was somewhat inferior with a mean sensitivity of 84%. However, recall, earlier in this article we highlighted that predicting the most common outcome is a relatively easy task in that most times you will be correct! The trade-off for this high-sensitivity, in the case of the logistic regression model, is a very low specificity (less than 1%) in that this approach had almost no ability to detect entrants likely to fail at least one of their first two years of undergraduate study at first sitting. In contrast, the machine learning algorithm had a modest, though appreciable, specificity at 32%. In effect this meant that the model was able to predict roughly one third of those students likely to encounter academic difficulties in their first two years. The superior predictive performance of the machine learning algorithm is also reflected in higher PPV and NPVs. That is, of those students that are predicted to pass both the clinical years without difficulty the machine learning algorithm was correct 84% of the time, on average, compared to 81% in the case of the logistic regression model. Similarly, of those entrants predicted to fail at least one year at first attempt, the machine learning algorithm was correct around third of the time (33%) whilst this value was slightly lower at 29% for the logistic regression approach (though recall, in this latter model, very low absolute numbers of students in this category were predicted).

In order to appreciate what this might mean in practice the average values, related to the predictive ability of the models, were translated into absolute numbers of candidates. Thus we can show how many candidates were correctly or incorrectly identified in the test (validation) dataset, as being likely to fail at least one year in the first two years of medical school. These are presented in the usual format of a two by two contingency table. In this case the values were rounded to one decimal place as they represent the averaged numbers across the thousand runs of each model.

**Table 1.** The accuracy of the two modelling approaches (averaged over 1000 runs) when predicting which medical school entrants will pass the first two years of medical school with no academic failures. The indices were significantly different between models (on Kruskal-Wallis testing) at the p<0.0001> level.

Model	Mean AUC (SD)	Mean PPV (SD)	Mean NPV (SD)	Mean Sensitivity (SD)	Mean Specificity (SD)
Logistic Regression	0.619 (0.01)	0.810 (0.005)	0.293 (0.184)	0.997 (0.002)	0.005 (0.004)
XGBoost	0.659 (0.011)	0.840 (0.009)	0.326 (0.021)	0.842 (0.04)	0.320 (0.07)

Abbreviations: AUC, Area Under the Curve; PPV, Positive Predictive Value; NPV, Negative Predictive Value

**Table 2.** Approximate values, calculated from results from 1000 runs, of the number of students in the test (validation) dataset predicted to have passed the first two years of medical school at first sittings, according to the two modelling approaches used (traditional vs machine learning). Note- correct predictions are in bold.

Logistic Regression	Predicted Passed	Predicted Failed
Actual Passed	**2494**	8
Actual Failed	5867	**3**
	3081	11

Machine Learning	Predicted Passed	Predicted Failed
Actual Passed	**2107**	395
Actual Failed	401	**189**
	2508	593

As can be seen in table 2, the average accuracy observed for the logistic regression model was approximately 81%. However, this was achieved by almost exclusively predicting entrants that would pass both pre-clinical years without difficulty. The model had almost no ability whatsoever to predict which candidates were likely to fail at least one year. In contrast, the models derived through the machine learning approach, on average achieved an overall mean accuracy of only roughly 74%. However, this latter modelling approach did demonstrate an ability to predict candidates at risk of failure: out of approximately 590 students in each test data sample who had failed at least one the preclinical year the machine learning algorithm was able to detect just under 200 of them, on average (i.e. roughly one third).

The machine learning models were not interpretable as such. However they did produce an indication of the relative importance of each input ‘feature’ (predictor) when predicting the outcome. We noted that student A-level and GCSE performance (i.e. high school grades) were usually the most potent predictors of success, along with the average UKCAT score achieved by peers in a student’s medical school cohort. However, somewhat concerningly, ethnicity also frequently made it into the top five predictors.

## Discussion

In this article we have discussed some of the advantages and potential limitations of using a machine learning (‘predictive’) approach compared to a traditional, statistical (‘explanatory’) modelling one. Our findings are in keeping with previous literature comparing logistic regression to machine learning approaches when attempting to predict relatively uncommon outcomes ( Walsh, Ribeiro and Franklin, 2018). That is, machine learning tends to, on average, demonstrate superior predictive ability compared to equivalent classical statistical approaches. At first glance, the overall improved ability of our machine learning algorithm over the logistic regression appears modest (i.e. an AUC of 0.66 vs 0.62). However, when predicting uncommon or rare events this could mean the difference between a predictive approach which is useless in practice, and one which has some utility. Despite the practical challenges our predictive modelling attempts faced, machine learning was able to identify correctly, in an unseen test dataset, which medical school entrants were at increased risk of failing at least one preclinical year. Nevertheless, this increased predictive ability came at a price. Compared to a conventional statistical approach, model development and training was time consuming and relatively computationally intensive. If machine learning is to be implemented it needs to able to deliver a ‘return on investment’. That is, demonstrate a positive impact on real-world problems that cannot be achieved by simpler methods. Moreover, although the relative importance of each predictor variable was reported, the actual modelling process was too complex to be interpretable. This increases the risk that, were changes in the population tested to occur, rendering the model invalid, this may not be immediately apparent to end-users (i.e. selectors). The only way to address this would be to periodically re-validate the model on more recent test datasets, where both predictors and outcomes were available.

In order to place our findings in a selection context it is worthwhile to conduct a brief thought experiment; consider a scenario where an admissions tutor had two relatively similar candidates that they were about to make an offer of study to. If the routinely collected data on both candidates were fed through the algorithm then the tutor would have the machine learning prediction at hand, to help support decision-making. Thinking back to the negative and positive predictive value of the machine learning model (as outlined in tables 1 and 2), if the algorithm suggested that the candidate was unlikely to fail an exam in the first two years then, in practice, the risk of this outcome would be, on average, roughly 15%. If, on the other hand, the algorithm predicted at least one failed year at first attempt then the risk would be approximately 33% (i.e. more than double of the former applicant). Given the high competition ratios normally encountered in medical school applications an admissions tutor may wish to take this into account if they had a plentiful supply of good quality candidates to select from. That is to say, they may wish not to make an offer to the candidate who has more than twice the risk of failing one of the first two years in medical school of another applicant. In healthcare settings, the suggestions made by automated decision support tools are often over ridden by human clinicians, at times acting on their own intuitions ( Roshanov *et al.*, 2013). It is not known whether this would be the case with analogous ‘selection-support’ tools.

In terms of strengths of this illustrative study, we had a relatively large dataset on which to train a machine learning algorithms. We also took, as far as was appropriate, a similar approach to model building and evaluation with the classical approach, using the logistic regression, in order to effect a fair comparison. In terms of limitations, there was a significant quantity of missing data for some of the predictor variables, which was dealt with through imputation, as well as iteration, in order to quantify the uncertainty that this introduced into the results. In the real world missing variables and information are very common, but it is not clear that, if the dataset were more complete, whether this would further advantage one modelling approach over the other. There was also information that was missing from a dataset that would normally be readily available to selectors, such as ratings from interviews.

Our selected example, though hopefully useful in showcasing the principles behind machine learning, was somewhat contrived. That is, in medical selection, there are clearly other important attributes that are evaluated above and beyond academic ability. It is well known that the manner in which a selection test is implemented affects the predictive validity ( Albanese, Farrell and Dottl, 2005). Therefore, even if usefully predictive algorithms were implemented into medical selection routinely, their ultimate impact on the demographics of the medical workforce would be partly determined by how they were used. As highlighted earlier, the use of such algorithms may have unintended consequences. In this case it is easy to foresee how an emphasis on academic ability may come at the price of rejecting other candidates, who have perhaps other qualities that they could bring to other aspects of medical education or clinical practice. Indeed, perhaps the greatest challenge in a medical selection scenario would be finding a suitable outcome target to train against. The lack of consensus over what constitutes a ‘good doctor’ has previously been referred to as ‘the criterion problem’ ( Cleland *et al.*, 2012). Even if there were agreement over these qualities then there are still the challenge of measuring these. Previous work in organisational psychology, including that related to medicine, has often relied on supervisor ratings as an outcome that can be used to validate selection measures ( Patterson *et al.*, 2017). However, this presents a number of challenges. Firstly, supervisor ratings, performance and actual clinical practice for that matter, would only be available after many years subsequent to the initial selection of medical school entrants. If a machine learning algorithm were to be used for new medical school applicants then the world may have changed since the original training data was used to create a machine learning algorithm. Thus the model may no longer be valid, or at least, not as accurate. Secondly, supervisor ratings would inevitably have an element of subjectivity in them and can be prone to bias ( Lefkowitz and Battista, 1995). It is known that machine learning algorithms, if trained on ratings based on human judgement, can actually exaggerate the very human biases that they intended to mitigate against (as illustrated in some of our initial examples provided). Thirdly, it is recognised that supervisor or peer ratings tend to be only able to discriminate between extreme characteristics, though ranking candidates may address this issue to some extent ( Goffin *et al.*, 1996). This, however, may not be an unsurmountable problem in practice if the role of a machine learning algorithm was to predict those in extreme groups.

It was of concern that ethnicity regularly featured in the top five predictors during model runs. This hints that such an algorithmic approach could, as highlighted in the examples given in the introduction, result in ethnic bias during selection. One way of mitigating this, at least to some extent, would be to remove ethnicity as a variable in the training data. If a machine learning approach did indeed enhance selection then this should ultimately be evidenced by a more effective, yet acceptably diverse, medical workforce in areas using such systems.

## Conclusion

Certainly it seems that machine learning and artificial intelligence are here to stay and will increasingly become part of our home and working lives. If this is to be the case then it is important that end-users are aware of both the strengths and limitations of this approach, as well as the unintended consequences that can result when carelessly implemented. In the case of medical selection there may be indeed be a role for machine learning to help support the decisions of selectors, faced with challenging choices between relatively homogenous, high performing candidates. However, if these methods are to result in an enhanced medical workforce then efforts should be made to develop datasets which includes suitable outcomes to train these models with. In the absence of these, the use of artificial intelligence in this setting runs the risk of exaggerating the very biases that machines were intended to eliminate.

## Take Home Messages

• On average, machine learning approaches tend to demonstrate superior predictive ability compared to equivalent conventional statistical approaches
• Prediction using machine learning could help support medical selectors decide between relatively homogenous, high performing candidates
• However, machine learning models have a number of key weaknesses and are only as good as the datasets they are trained on
• Machine algorithms wouldn’t automatically accommodate changing trends, and therefore may become less valid without a user being aware of this
• If carelessly implemented machine learning algorithms can mimic or even exaggerate human biases

## Notes On Contributors

Paul Tiffin is a Reader (Associate Professor) in Psychometric Epidemiology at the University of York and a National Institute for Health Research Career Development Fellow. His main research interest is the development and application of statistical and psychometric modelling approaches.

Lewis Paton is a Research Fellow (Assistant Professor) at the University of York. He is a statistician interested in developing novel methodological approaches.
