# Supplementary material for: Artificial or intelligent? Machine learning and medical selection: possibilities and risks
Source: MedEdPublish (2016). 2018 Nov 13;7:256. [Version 1] doi: 10.15694/mep.2018.0000256.1 (PMC10712013; doi:10.15694/mep.2018.0000256.1)
Supplement: Peer review history

# Reviewer report: Reviewer response for version 1

**DOI:** 10.21956/mep.19707.r29481

**Published:** 2018-11-26

**Authors:** Mark Hanson

**Affiliations:** 1 Hospital for Sick Children

This review has been migrated. The reviewer awarded 4 stars out of 5 This paper introduces readers to the unfolding field of Artificial Intelligence (AI) and its potential for application to medical school admissions. Myself as a reader not familiar (but very interested) in AI it provides basic knowledge that will provide readers such as myself with important information. The authors also opine as to possibility that use of AI within admissions and its role in selecting the future healthcare professional workforce should be considered a health technology. As such this designation will bring to bear government regulations with respect to development and implementation – that warrants consideration. For instance, a caution the paper illuminates is the ‘black box’ element of AI and the risk of continuing human biases already present within admissions with these new technologies. The authors generate more questions than answers – which I believe was their intent – and the need for the field to come together in a forum to discuss and debate the future use of AI within medical school admissions.

Reviewer Expertise:

NA

No decision status is available

# Reviewer report: Reviewer response for version 1

**DOI:** 10.21956/mep.19707.r29477

**Published:** 2018-11-21

**Authors:** Barbara Griffin

**Affiliations:** 1 Macquarie University

This review has been migrated. The reviewer awarded 3 stars out of 5 This paper pushes our thinking into new territory, providing both an introduction on how machine learning might be applied to selection as well as data to illustrate its utility. The possibility for non-linear combinations of data has potential given that most of our current practice uses a linear combination or a sequence of hurdles, neither of which can account for complex profiles of scores.However, as highlighted by the authors, academic failure in preclinical years is a relatively rare occurrence, which could suggest current processes are actually relatively successful. The results section was a little light on detail so I was also left wanting to know more information, for example on the top five predictors. This served to highlight the problem of the ‘black box’! Given that ethnicity and other unchangeable factors, such as socioeconomic status, could not be used to reject applicants, and that there is little variance in selected applicants’ academic ability, it is possible that machine learning has more to offer as a post-selection means of identifying ‘at risk’ students for targeted support rather than to make selection decisions.

Reviewer Expertise:

NA

No decision status is available

# Reviewer report: Reviewer response for version 1

**DOI:** 10.21956/mep.19707.r29479

**Published:** 2018-11-20

**Authors:** Jennifer Cleland

**Affiliations:** 1 University of Aberdeen

This review has been migrated. The reviewer awarded 4 stars out of 5 I read this paper with interest as it brings a very different perspective to an old problem. I was particularly interested in the sections which reflect on the potential "black box" issue of machine learning decision making and not being able to explain to applicants/candidates why they had been rejected. Would this be acceptable or not?The issue of little variance between medical school applications is also of interest - is there enough information for the machine to make decisions or not? The authors then test out machine learning, entering academic performance, etc, into the machine but also adding in some "contextual data" (e.g., socio-economic class: SEC) . This is very topical in the UK at least. I was relieved to see that SEC was not a predictor, and this has face validity given other studies (e.g., Kumwenda et al., 2017).Sadly, the machine learning outcomes were still not very great, with reasonably weak specificity and sensitivity (but better than nothing as the authors point out). I welcomed this paper as it opens a whole new discussion in the field.

Reviewer Expertise:

NA

No decision status is available

# Reviewer report: Reviewer response for version 1

**DOI:** 10.21956/mep.19707.r29480

**Published:** 2018-11-18

**Authors:** Ken Masters

**Affiliations:** 1 Sultan Qaboos University

This review has been migrated. The reviewer awarded 3 stars out of 5 A useful paper for introducing the topic, but I wonder if the paper attempts to do too much, and this results in a loss of focus – is it a discussion of machine learning in general, or is it reporting on research? It is listed as a personal view, but then reports on research. It would have been better if the paper had been classified as a research article, and the introductory material adjusted a little to conform more closely to the research article style, and then the researchers report on their research. In this way, the researchers could keep virtually all of their paper, but position it not so much as commenting on machine learning, but on their research. The conclusion would also need to be appropriately amended.Some minor points:• Some very long paragraphs that could be broken up in order to ease the reading experience, especially with such a complex topic. Similarly, the paper could benefit from sub-headings to help to sign-post the path as the reader works through it.• A few typos (“Tradiotional”)

Reviewer Expertise:

NA

No decision status is available

# Reviewer report: Reviewer response for version 1

**DOI:** 10.21956/mep.19707.r29478

**Published:** 2018-11-14

**Authors:** Ian Wilson

**Affiliations:** 1 International Medical University

This review has been migrated. The reviewer awarded 4 stars out of 5 This is a very interesting article that helps the reader to have a greater understanding of machine learning and artificial intelligence. The research was comprehensive, but like so many studies involving modelling it was somewhat artificial. However as a demonstration of the strengths and weaknesses of the machine learning approach it will prove valuable to researchers interested in this area. I am sure we will see increasing research in medical education that involves machine learning and AI and selection is one area that will be amenable to such an implementation.I congratulate the authors for providing a readable paper in such a complex area.

Reviewer Expertise:

NA

No decision status is available
